# The Janus face of rosiglitazone

**DOI:** 10.18632/oncotarget.26532

**Published:** 2018-12-28

**Authors:** Christine Pich, Liliane Michalik

**Affiliations:** Center for Integrative Genomics, University of Lausanne, Lausanne, Switzerland

**Keywords:** rosiglitazone, PPARγ, melanoma, tumor microenvironment

**Thiazolidinediones: anti-diabetic drugs to treat cancer?**

Thiazolidinediones (TZDs) are potent insulin sensitizers used to prevent and treat type 2 diabetes [1]. Widely-used in the early 2000’, pharmacological treatments with these compounds have been drastically reduced due to adverse effects, like weight gain, hepatotoxicity, and congestive heart failure [1, 2]. Nevertheless, two members of this family of compounds, rosiglitazone (Avandia) and pioglitazone (Actos), are still approved for the treatment of type 2 diabetes in selected patients and under restricted conditions.

Besides treatment of diabetes, TZDs have been proposed as a therapeutic option for cancer. This was initially based on the observation that the nuclear receptor PPARγ -the best characterized molecular target of TZDs-exhibits anti-cancer properties. PPARγ is involved in the regulation of several conditions that are key for controlling tumor growth, like cell proliferation, cell survival, cell differentiation, and inflammation [2, 3]. Moreover, genetic evidence shows that loss of PPARγ function predisposes to colon, breast, ovary and skin cancers [4]. Finally numerous studies have reported anti-tumoral effects of PPARγ activation both *in vitro* and in *in vivo* mouse models, possibly also in humans [4]. Despite anti-cancer properties reported in many cancer cell types, clinical trials involving TZDs and activation of PPARγ, which were performed over the past 15 years have been disappointing, showing little therapeutic efficacy [2].

**Undesirable pro-tumorigenic actions of rosiglitazone in metastatic human melanoma cells.**

Skin melanoma, an aggressive malignant neoplasm of melanocytes, is the most dangerous form of skin cancer. Although it represents only 4% of all skin cancers, it is currently responsible for 80% of skin cancer-related deaths. Cancer research has taught us that melanoma require multiple therapeutic approaches. The search for treatments against melanoma is therefore still active, despite the recent breakthrough of immuno- and molecularly targeted therapies [5].

**Figure 1:**
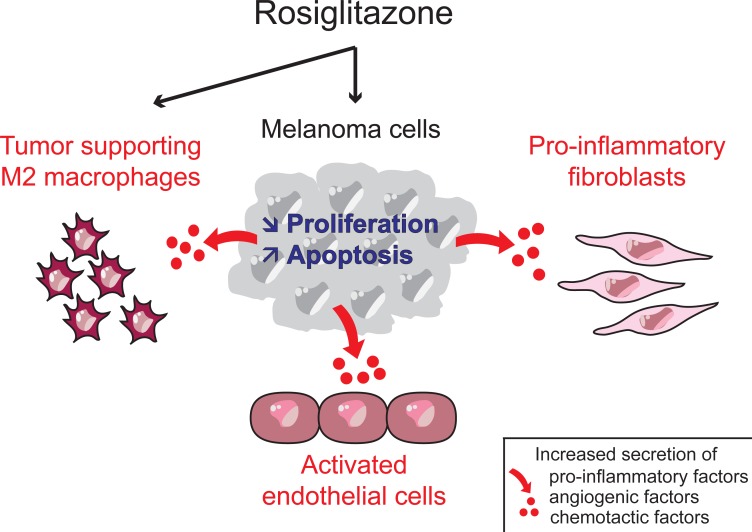
The Janus face of rosiglitazone in melanoma On the one hand, rosiglitazone shows cell-autonomous anti-cancer actions (in blue) in melanoma cells, by preventing proliferation and promoting apoptosis [6]. On the other hand, rosiglitazone shows pro-cancer actions (in red). These include cell-autonomous impact on macrophages [6], as well as paracrine effects on fibroblasts, endothelial cells and tumor associated macrophages [7]. Of note, while the metabolic actions of rosiglitazone are clearly mediated by PPARγ, its actions on cancer cells can only partially be explained by PPARγ activation [2]. The figure was inspired by discussion with Dr Thanh Nhan N'Guyen.

Several studies conducted over the past 20 years supported the use of TZDs and of PPARγ activation as a therapeutic option to treat melanoma [6]. However, these studies showed anti-cancer effects on the malignant cells (Figure 1), while they neglected the role played by non-malignant cells of a tumor microenvironment. Recently, a study of TZDs impacts extended beyond the melanoma cell itself, and identified a new pro-tumorigenic paracrine action of rosiglitazone (RGZ) [7]. This pro-tumorigenic action of RGZ involves increased secretion of cytokines, chemokines and angiogenic factors by a subset of human melanoma cells, in turn activating non-malignant fibroblasts, endothelial cells and macrophages in a tumor-friendly way (Figure 1). In mice, tumors derived from human melanoma cells and exposed to RZG exhibited accelerated development, and increased inflammation and angiogenesis. In this model of melanoma, RGZ pro-tumorigenic action prevailed over its anti-cancer action [7]. In addition, in line with these results, RGZ retained this pro-tumorigenic effect on human melanoma cells exposed to Vemurafenib, a recently approved pharmacological treatment of melanoma targeting the BRAF mutant BRAF^V600E^ [8] [Pich et al, unpublished results]. In these melanoma cells, RGZ partially prevented Vemurafenib from inhibiting proliferation and interleukins secretion, thereby reducing Vemurafenib efficacy.

Collectively, these data suggest that adverse consequences may result from exposing patients with existing melanoma to RGZ, and potentially to other PPARγ agonists. Finally, although focusing on melanoma, the study by Pich and collaborators suggests that RGZ may have similar undesirable impacts in other pre-existing tumors, like non-melanoma skin cancers and bladder cancers [7].

**Thiazolidinediones as an option to treat cancer: balancing pro-tumorigenic and anti-cancer effects.**

The vast majority of studies conducted on cohorts of diabetic patients with no pre-existing tumors report overall preventive benefits of TZDs, with the exception of pioglitazone, which was suspected to slightly increase the risk of bladder cancers, non-Hodgkin lymphoma and melanoma [2, 9]. For existing tumors, investigation of TZDs as a therapeutic option led to contradictory findings, showing pro-tumorigenic effects on the one hand and anti-cancer effects on the other hand. These contrasting results seem to result from a combination of complex PPARγ-dependent or -independent impacts, context-and compound- specific actions and, as shown in [7], pro-tumorigenic effects on the tumor microenvironment opposing the anti-cancer effects observed on the malignant cells (Figure 1). Recent progress in omics and single cell techniques will likely allow for a better understanding of how the various cells types in a tumor respond to TZDs.

TZDs complex and contrasting actions make it impossible to generalize the impact of pharmacological treatment in regard to cancer development. Moreover, they partially explain why promising *in vitro* studies have not been translated into convincing clinical trials. Besides TZDs, the search for novel PPARγ agonists is still active [10] and their impact on cancer cells is still questioned. Finding PPARγ agonists that retain anti-cancer activities but lack pro-tumorigenic properties would be potential therapeutic options for cancer treatment.
